# A Conceptual Framework for Managing Oral Intake in Head and Neck Cancer Patients Undergoing Chemoradiotherapy

**DOI:** 10.3390/nu18081180

**Published:** 2026-04-09

**Authors:** Marco Cintoni, Elena Leonardi, Pauline Celine Raoul, Silvia Longo, Mariangela Massaccesi, Marta Palombaro, Gabriele Egidi, Francesco Pastore, Emanuele Rinninella, Esmeralda Capristo, Antonio Gasbarrini, Maria Antonietta Gambacorta, Maria Cristina Mele

**Affiliations:** 1 UOC di Nutrizione Clinica, Dipartimento di Scienze Mediche e Chirurgiche, Fondazione Policlinico Universitario A. Gemelli IRCCS, Largo A. Gemelli 8, 00168 Rome, Italy; leonardielena712@gmail.com (E.L.); marta.palombaro@guest.policlinicogemelli.it (M.P.); gabriele.egidi@policlinicogemelli.it (G.E.); emanuele.rinninella@unicatt.it (E.R.); mariacristina.mele@unicatt.it (M.C.M.); 2 Centro di Ricerca e Formazione in Nutrizione Umana, Università Cattolica del Sacro Cuore, 00168 Rome, Italy; esmeralda.capristo@unicatt.it (E.C.); antonio.gasbarrini@unicatt.it (A.G.); 3 UOC Radioterapia, Dipartimento di Diagnostica per Immagini, Radioterapia Oncologica ed Ematologia, Fondazione Policlinico Universitario A. Gemelli IRCCS, Largo A. Gemelli 8, 00168 Rome, Italy; silvia.longo@policlinicogemelli.it (S.L.); mariangela.massaccesi@policlinicogemelli.it (M.M.); francesco.pastore@policlinicogemelli.it (F.P.); mariaantonietta.gambacorta@policlinicogemelli.it (M.A.G.); 4 Dipartimento di Medicina e Chirurgia Traslazionale, Università Cattolica del Sacro Cuore, 00168 Rome, Italy; 5 UOS Medicina della Grande Obesità, Dipartimento di Scienze Mediche e Chirurgiche, Fondazione Policlinico Universitario A. Gemelli IRCCS, Largo A. Gemelli 8, 00168 Rome, Italy; 6 UOC Medicina Interna e Gastroenterologia, Dipartimento di Scienze Mediche e Chirurgiche, Fondazione Policlinico Universitario A. Gemelli IRCCS, Largo A. Gemelli 8, 00168 Rome, Italy; 7 Dipartimento di Scienze Radiologiche ed Ematologiche, Università Cattolica del Sacro Cuore, 00168 Rome, Italy

**Keywords:** head and neck cancer, nutritional impact symptoms, nutritional intervention, dysphagia, xerostomia, oral intake

## Abstract

Patients with head and neck cancer (HNC) face a high risk of malnutrition and sarcopenia, often exacerbated by the toxicities of chemoradiotherapy, such as dysphagia, xerostomia, and mucositis. These Nutritional Impact Symptoms significantly compromise oral intake and negatively affect quality of life. This paper presents a conceptual framework designed to support clinicians in optimizing oral intake through personalized nutritional management. Central to this approach is the integration of systematic screening using MUST, the Malnutrition Universal Screening Tool (MUST), and the Nutritional Risk Screening 2002 (NRS-2002). Furthermore, functional assessment of swallowing via instrumental studies (VFSS/FEES) is essential for tailoring dietary textures according to the International Dysphagia Diet Standardization Initiative framework. Key nutritional strategies include high-energy and high-protein oral fortification, the use of oral nutritional supplements, and specific dietary adjustments addressing pain management and sensory alterations. A multidisciplinary approach involving nutritionists, speech-language pathologists, and oncologists is paramount to transition from reactive symptom management to proactive “adaptive nutrition,” ultimately improving clinical outcomes and patient survival.

## 1. Introduction

Head and neck cancer (HNC) encompasses a heterogeneous group of malignant tumors arising in the upper aerodigestive tract. It has garnered increasing attention due to its epidemiological significance and diverse etiology, particularly among major subsites including the oral cavity, oropharynx, larynx, and hypopharynx. According to the Global Cancer Observatory, HNC represents the seventh most prevalent cancer worldwide, with approximately 660,000 new cases diagnosed and an estimated 325,000 deaths reported annually [1,2]. The epidemiological landscape varies significantly by subsite. The oropharynx is characterized by the duality of HPV-positive and HPV-negative cancers; studies indicate that HPV-positive oropharyngeal squamous cell carcinoma is experiencing a significant rise in incidence, particularly among younger male populations, diverging from the traditional risk factor profile dominated by alcohol and tobacco use [3,4]. Conversely, the larynx, accounting for approximately 20% of HNC diagnoses, shows stabilization or slight increases in incidence rates across various populations, where tobacco remains a primary contributory factor [1,5]. The hypopharynx typically presents an aggressive clinical course and accounts for a smaller proportion of cases, with advanced stage diagnosis often leading to poorer prognostic outcomes [5]. While the epidemiological landscape is complex and varies by subsite, the high prevalence of malnutrition remains a critical and pervasive challenge across all HNC diagnoses. The prevalence of malnutrition among patients with HNC is alarmingly high, presenting significant challenges in the management of these patients [6]. Studies have reported that over 50% of HNC patients exhibit signs of malnutrition even before the onset of treatment, often attributed to factors such as cancer-related dysphagia, inadequate oral intake, and the complex interactions of tumor biology with nutritional status [7]. A recent longitudinal study revealed that the risk of malnutrition escalated significantly over the course of treatment, with rates jumping from 23.3% at the beginning of therapy to as high as 78.7% toward treatment completion, highlighting the critical need for proactive nutritional assessments [8]. Furthermore, a comprehensive review highlighted that malnutrition directly correlates with advanced tumor stage and the severity of treatment-associated side effects, such as radiation-induced mucositis [7,9].

This underscores the vulnerability of HNC patients to nutritional deficiencies, which, if left unaddressed, can adversely impact treatment outcomes and quality of life [10,11]. Linked to malnutrition is sarcopenia, defined as the loss of skeletal muscle mass and strength. The incidence rates of sarcopenia in HNC patients have been shown to vary widely, ranging from 6.6% to 64.6% [12]. This wide disparity reflects differences in study methodologies and populations.

Sarcopenia is particularly concerning because it is associated with poorer prognosis, diminished response to therapy, and heightened risk for postoperative complications [13,14,15]. The interplay between malnutrition and sarcopenia further complicates the clinical picture, as inadequate nutritional intake is a key contributor to muscle wasting [12,16]. Hence, implementing comprehensive nutritional interventions based on early screening and assessment is essential to enhance treatment tolerance and overall survival [11,14]. Despite the well-documented prevalence of these issues, there remains a clinical gap in standardizing proactive, comprehensive dietary strategies that merge symptom management with swallowing preservation. To address these complex challenges, clinical practice must shift from a static dietary prescription to a dynamic model of “Adaptive Nutrition”. Unlike conventional nutritional interventions, which typically react to weight loss or established malnutrition with standardized and static supplementation, Adaptive Nutrition anticipates the predictable physiological decline associated with chemoradiotherapy [8,9]. “Adaptive Nutrition” is defined as a continuous, dynamic model of nutritional support that adjusts in real time to the patient’s evolving functional and symptomatic status during treatment, drawing on continuous clinical surveillance rather than merely reacting to established malnutrition. This proactive strategy integrates continuous clinical surveillance, using validated screening tools and diagnostic criteria for malnutrition, with real-time functional adjustments based on instrumental swallowing assessments; moreover, by systematically modulating food texture according to the IDDSI framework, regulating temperature to minimize mucosal irritation, and enhancing nutrient density in response to evolving toxicities, clinicians can preserve the patient’s oral intake capacity for as long as possible. This approach not only aims to meet caloric and protein targets but also strives to maintain the functional integrity of the swallowing musculature, thereby mitigating the severity of sarcopenia and potentially improving long-term rehabilitation outcomes.

## 2. Materials and Methods

This work was developed as a narrative review and expert opinion to design a conceptual framework for HNC patients undergoing chemoradiotherapy. A structured literature search was conducted in PubMed and Web of Science databases, focusing on publications from 2000 to 2024 that addressed nutritional screening, swallowing assessment, and dietary interventions in HNC. The search strategy utilized the following Boolean string: (“head and neck cancer” OR “HNC”) AND (“chemoradiotherapy” OR “radiotherapy”) AND (“dysphagia” OR “nutritional impact symptoms” OR “xerostomia”) AND (“nutritional support” OR “dietary intervention”). Relevant guidelines, including ESPEN recommendations and IDDSI framework documents, were also considered. Articles were initially screened based on title and abstract, followed by a full-text review for relevance to oral intake optimization. The inclusion criteria were studies involving adult HNC patients that reported nutritional outcomes or interventions related to oral intake and were published in English. Exclusion criteria were pediatric populations, surgical-only treatments, and studies without dietary endpoints. To complement the literature review, a multidisciplinary panel contributed to the synthesis of practical recommendations. As this is a narrative review lacking a formal structured Delphi process, the proposed recommendations represent an expert-guided conceptual integration of existing high-level guidelines (e.g., ESPEN, IDDSI) and best-practice observational data. To clarify the positioning of this framework and enhance clinical reliability, each recommendation was explicitly evaluated to distinguish between reorganizations of existing, high-level evidence-based guidelines and new clinical proposals derived from expert consensus.

## 3. The Double-Edged Sword of Treatments in HNC

Standard treatment for HNC involves radiotherapy, chemotherapy, and surgical interventions. While therapeutically effective, the secondary complications of these regimens often result in substantial physical and psychological distress. Nutritional Impact Symptoms (NIS), a term emphasized in the ESPEN guidelines, describe treatment-related toxicities (e.g., dysphagia, xerostomia, mucositis) that compromise oral intake and contribute to malnutrition and sarcopenia in cancer patients. Dysphagia remains a prevalent and debilitating complication of both radiotherapy and surgical intervention. Similarly, radiation-induced salivary gland dysfunction frequently manifests as xerostomia and dysgeusia, which typically lead to food aversion and reduced energy intake, while systemic effects such as nausea and emesis further complicate the clinical picture [17,18,19,20,21,22,23]. These challenges are often exacerbated by mucositis, in which painful ulcerations serve as a primary barrier to adequate dietary intake [24,25,26]. NIS as previously mentioned, are prevalent among patients with HNC. These conditions often necessitate modifications in food texture, prompting patients to choose softer foods or purees, thus limiting the variety and nutritional value of their diets [24]. Additionally, mucositis presents serious challenges, with many patients experiencing painful sores in their mouths and throats as a direct consequence of chemotherapy and radiation. This inflammatory condition affects approximately 60% of HNC patients and can lead to extreme discomfort while swallowing, drastically influencing meal times and overall food intake [27]. The pain associated with mucositis can deter patients from eating, ultimately leading to inadequate caloric and nutrient consumption.

## 4. Nutritional Assessment

A comprehensive nutritional assessment is critical for the effective management of malnutrition in patients with HNC. At the time of diagnosis, the implementation of mandatory screening tools such as the Malnutrition Universal Screening Tool (MUST) and the Nutritional Risk Screening 2002 (NRS-2002) is essential for identifying individuals at risk of malnutrition [28]. These tools serve as a preliminary step in recognizing patients who require further nutritional evaluation and intervention. While both tests are valuable, a recent study found that the NRS-2002 correlated more strongly with the GLIM (Global Leadership Initiative on Malnutrition) criteria, identifying a greater number of patients at risk of malnutrition [28]. Following the initial screening, the GLIM criteria provide a standardized method for diagnosing malnutrition [29] (Level of Evidence: High, based on validated guidelines). This dual-component approach requires the presence of at least one phenotypic criterion and at least one etiological criterion. The phenotypic factors include specific, quantifiable metrics such as non-volitional weight loss (e.g., >5% within 6 months or >10% beyond 6 months), low Body Mass Index (BMI, with population-specific cut-offs), and reduced muscle mass (confirmed by imaging or validated tools). Etiological components emphasize underlying factors such as inflammation (acute or chronic disease-related), and reduced food intake or assimilation (e.g., <50% of energy requirements for >1 week) [30]. The integration of these parameters allows for a multifaceted approach to diagnosing malnutrition, ensuring that healthcare professionals can accurately identify individuals at varying risk levels [31]. Research indicates that adopting a systematic nutritional assessment incorporating both mandatory screening tools and GLIM criteria not only improves the identification of malnutrition but also enhances the prognostic evaluation of treatment response, clinical outcomes, and quality of life in HNC patients [32,33].

### 4.1. Functional Assessment of Swallowing

In the management of dysphagia within the context of HNC, the functional assessment of swallowing is essential for establishing an effective treatment strategy. While the primary diagnosis of dysphagia is conducted by speech-language pathologists (SLPs) using standardized protocols [34], the clinical nutritionist plays a critical role in integrating the findings from instrumental assessments—specifically Videofluoroscopic Swallow Studies (VFSS) and Fiberoptic Endoscopic Evaluation of Swallowing (FEES)—into the nutritional care plan [35]. These assessments allow for a comprehensive evaluation of swallowing function, revealing physiological impairments that can significantly impact nutritional intake and overall health [36]. The clinical nutritionist’s expertise is vital when analyzing the VFSS and FEES results, as they can correlate the observed swallowing mechanics with appropriate dietary modifications. This collaborative approach ensures that patients are placed on the correct dysphagia diet, based on evidence obtained from the instrumental assessments. For instance, data derived from VFSS allows clinicians to determine the safest food textures and fluid viscosities, aligning recommendations with the IDDSI framework [37,38]. This standardized framework provides clear guidelines to classify food textures and fluid consistencies, establishing baseline IDDSI levels that align with the patient’s swallowing capabilities [39]. By collaborating closely with SLPs and utilizing the insights gained from instrumental assessments, clinical nutritionists contribute significantly to the dietary management of dysphagia [34]. This partnership facilitates the creation of dietary plans that not only ensure nutritional adequacy but also promote safe swallowing practices tailored to the patient’s individual needs [40].

### 4.2. Nutritional Intervention

#### 4.2.1. Proactive Counseling and Macronutrient Targets

In the context of nutritional intervention, proactive dietary counseling is essential, particularly given the specific recommendations outlined in the ESPEN guidelines [35], stipulating that oncologic patient, including those with HNC, should achieve daily protein targets of 1.2 up to 2.0 g per kilogram of body weight, depending on their overall nutritional status and phase of treatment [41] (Level of Evidence: High).

For instance, during chemotherapy or radiation therapy, patients often require higher protein intakes to support muscle preservation and repair, ideally reaching the upper end of this protein spectrum [37]. Concurrently, ESPEN recommends tailoring total caloric intake individually, generally targeting a range of 25 to 30 kcal/kg/day to ensure energy adequacy and weight maintenance [38,41]. Meeting these energy and protein requirements is critical for mitigating the risk of malnutrition driven by treatment-related toxicities.

#### 4.2.2. Texture Modification and Muscle Preservation

Preserving oral muscle function in patients with dysphagia also necessitates a careful approach in food texture selection [38]. It is critical to offer a variety of textures and consistencies, aligned with the patients’ swallowing capabilities as assessed through instrumental evaluations such as VFSS and FEES. Clinical nutritionists should use the IDDSI framework to categorize foods and fluids, thereby facilitating safe oral intake while encouraging the continued engagement of oral musculature [40,42] (Level of Evidence: High).

Varied textures help to ensure that swallowing remains a functional task rather than a stressful challenge, thereby promoting oral health and comprehensive nourishment [43].

#### 4.2.3. Medical Nutrition Therapy

Despite intensive and aggressive oral interventions, many HNC patients may be unable to meet nutritional requirements via the oral route alone. When oral intake remains inadequate (typically <60% of estimated needs for more than 1–2 weeks) or is deemed unsafe, ESPEN guidelines recommend initiating Medical Nutrition Therapy [42]. Enteral Nutrition (EN)—delivered via nasogastric (NG) tubes or percutaneous endoscopic gastrostomy (PEG)—is the preferred modality as it maintains gut integrity and immune function [42]. However, the presence of a feeding tube should not preclude oral intake; rather, it should be utilized as a complementary approach while encouraging “therapeutic tastings” or minimal oral intake to prevent disuse atrophy of the swallowing muscles [44]. Parenteral Nutrition (PN) is generally reserved for cases in which the gastrointestinal tract is nonfunctional or when EN is contraindicated or insufficient to meet metabolic demands [38,45].

## 5. Dietary Management of Dysphagia and Odynophagia

### 5.1. Progression of Texture Modification

The progressive modification of dietary texture is a cornerstone in the management of dysphagia and odynophagia during the acute phase of HNC treatment. Following the IDDSI framework [46], dietary adjustments must be methodically applied to enhance swallowing safety and overall nutritional adequacy for patients exhibiting difficulties. At the highest texture levels designated for individuals without significant swallowing difficulties, indicated as Level 7 (Regular) and Level 6 (Soft and Bite-sized), tips for maintaining lubrication and moisture retention are essential.

**Figure 1:**
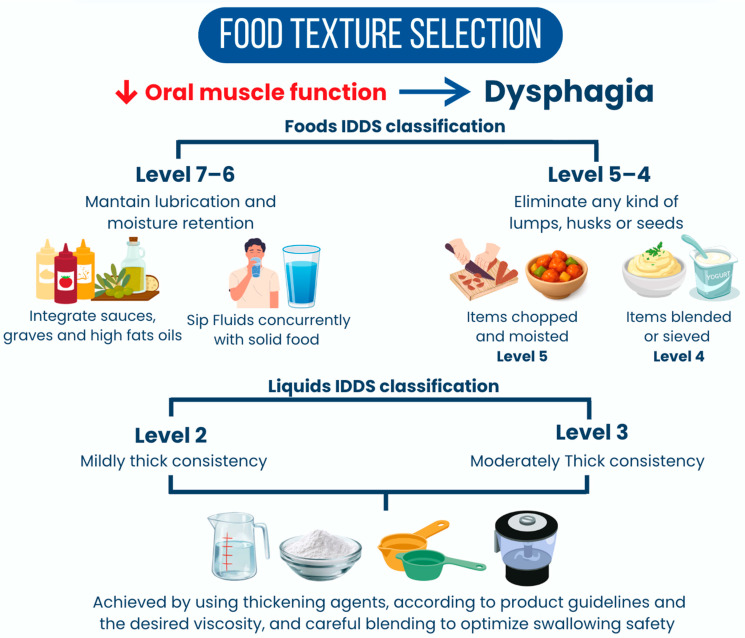
IDDSI Framework Application.

Clinical nutritionists are encouraged to integrate sauces, gravies, and high-fat oils into meals to improve moisture levels. These modifications not only enhance flavor and palatability but also help create a more cohesive food bolus. Additionally, encouraging patients to sip fluids concurrently with solid food can facilitate easier swallowing and prevent dryness, which could exacerbate odynophagia [47,48]. As healthcare providers navigate the progression from minced and moist to puréed textures, indicated as Level 5 (Minced and Moist) to Level 4 (Puréed), they should emphasize specific techniques for preparing foods to eliminate lumps, husks, or seeds that can pose choking hazards. For Level 5 foods, minced items should be finely chopped and moistened sufficiently to maintain cohesiveness without being overly runny. Transitioning to Level 4, foods must be expertly blended or sieved to achieve a smooth consistency free from lumps or granularity (Figure 1). Using high-powered blenders can help ensure compliance with IDDSI criteria, allowing patients to safely consume puréed diets that meet both nutritional needs and texture standards [48,49,50].

In addition, appropriate management of fluid consistency is also critical in dysphagia treatment. Strategies for achieving IDDSI Level 2 (Mildly Thick) and Level 3 (Moderately Thick) fluids involve using specific thickening agents that effectively alter the viscosity of liquids while ensuring they remain palatable, as detailed in the lower panel of Figure 1 [51]. For Level 2 fluids, a mildly thick consistency can be achieved by using thickening agents according to product guidelines, which specify a particular thickener-to-liquid ratio based on the desired viscosity. Similarly, for Level 3 fluids, achieving moderate thickness requires careful measurement and blending to optimize swallowing safety. Practicing visual and tactile assessments of fluid consistency, such as the spoon-tilt test, is advised to ensure the fluid does not run too quickly while still flowing easily when swallowed. These techniques are vital for preventing aspiration and ensuring that patients can hydrate safely despite dysphagia [52,53,54].

### 5.2. High-Energy, High-Protein (HE/HP) Oral Fortification

#### 5.2.1. Nutrient Density Maximization

High-energy, high-protein (HE/HP) oral fortification plays a critical role in increasing nutrient density without significantly increasing food volume. This approach is particularly vital for patients who require texture-modified diets, as it ensures adequate caloric and protein consumption to avoid malnutrition and promote recovery [55,56].

To optimize caloric density without exceeding the patient’s volume tolerance, several strategies can be employed with readily available ingredients. One effective method is to add skim milk powder or cream cheese to foods and beverages. Both components are rich in protein and contribute significant calories without markedly expanding the serving size. For instance, incorporating just a few tablespoons of skim milk powder can add approximately 25–30 g of protein and 150–200 calories to a smoothie or soup, effectively enhancing the nutritional profile [55]. Another strategy is to use neutral-tasting oils, such as canola or olive oil, which can seamlessly blend into different foods while providing a concentrated source of calories. These oils can be drizzled over steamed vegetables or mixed into purees, adding about 90–120 calories per tablespoon without altering the taste [57]. Additionally, fortified protein powders are highly effective for patients struggling to meet their protein needs. These powders can easily be mixed into a variety of foods, such as yogurt, oatmeal, or smoothies, to boost protein intake by 20–30 g per serving, depending on the product used [56]. Crucially, these nutritional enhancements must be implemented in alignment with the IDDSI framework, ensuring that fortification does not compromise texture safety. Products thickened to meet IDDSI Level 2 or Level ?3can be fortified without compromising the safety and swallowability of meals. For instance, using commercially available thickeners can create the desired viscosity to keep fluids and foods at an appropriate consistency, enabling patients to enjoy calorically dense meals that are safe to swallow [57,58]. These strategies exemplify a comprehensive approach to dietary management for patients with dysphagia and emphasize that nutrient density maximization through thoughtful ingredient selection can substantially improve nutritional outcomes [55,57].

#### 5.2.2. Oral Nutritional Supplements (ONS)

In the dietary management of dysphagia and odynophagia, the integration of HE/HP oral fortification must also consider the role of Oral Nutritional Supplements (ONS). These supplements play a significant role in ensuring patients meet their nutritional needs when fortified foods alone are insufficient [59,60]. Standard polymeric ONS provide a balanced source of macronutrients (carbohydrates, proteins, and fats) and can effectively augment caloric and protein intake for dysphagic patients. They are generally well-tolerated and can be customized with flavorings or thickeners to meet individual preferences and adhere to the IDDSI guidelines [61]. In contrast, disease-specific ONS are formulated to address particular nutritional needs based on the underlying condition. For instance, ONS containing eicosapentaenoic acid (EPA) and docosahexaenoic acid (DHA), which are omega-3 fatty acids, have been explored for their potential anti-inflammatory benefits and positive effects on immune function. However, the evidence regarding their efficacy is mixed; while some studies suggest they may offer therapeutic benefits for patients with certain conditions, others report inconclusive outcomes [61,62]. Consequently, clinicians should evaluate the appropriateness of these specialized formulations on a case-by-case basis, weighing individual patient factors against clinical goals.

**Figure 2:**
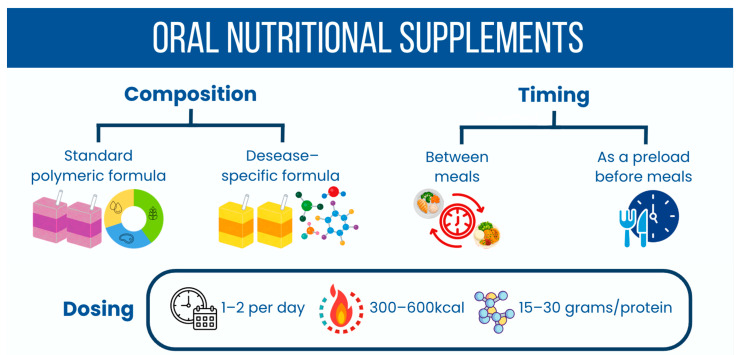
Oral Nutritional Supplements (ONS) recommendations.

The timing and dosing of ONS are critical for optimizing effectiveness without compromising meal intake. ONS should complement, not replace, regular meals, as they lack the complete range of flavors, textures, and physiological satisfaction that whole foods provide. To prevent the “displacement effect”—where supplements reduce appetite for subsequent meals—it is generally advisable to administer ONS between meals or strategically as a split dose throughout the day [63]. Recommendations often suggest providing ONS 1–2 times per day, adjusting based on caloric needs and individual tolerance, while ensuring that these supplements offer substantial energy and protein contents—aiming for approximately 300–600 kcal and 15–30 g of protein per serving (Figure 2), depending on the individual’s requirements [64]. ONS represent an essential component in the dietary management of patients with dysphagia and odynophagia, particularly when aligned with proactive nutritional strategies. Both standard polymeric and disease-specific formulations should be used judiciously, ensuring that they complement regular dietary intake through appropriate timing and dosing that maximizes nutritional status while respecting the individual’s preferences and swallowing capabilities [50,65].

Furthermore, the specific amounts of ONS and nutritional fortifications detailed here should not be viewed as a rigid or prescriptive guideline. Instead, they represent a baseline that requires careful, individualized adjustment based on the patient’s specific background. Clinicians must dynamically tailor these interventions by considering factors such as the patient’s baseline nutritional status, age, metabolic comorbidities (e.g., diabetes or renal impairment), and the specific severity and trajectory of their treatment-induced toxicities.

### 5.3. Pain Management-Driven Dietary Adjustments

**Figure 3:**
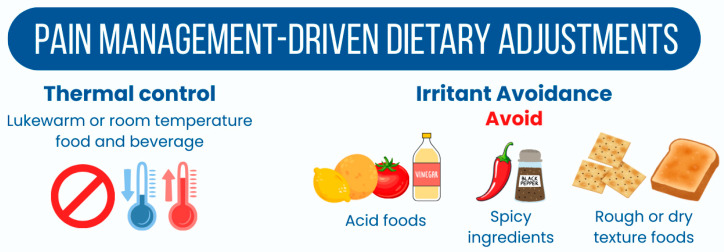
Pain Management-Driven Dietary Adjustments.

Pain Management-Driven Dietary Adjustments in the context of dysphagia and odynophagia necessitate careful consideration of food and fluid choices to alleviate discomfort associated with eating. Effective strategies center on two main pillars: thermal control and irritant avoidance, both of which significantly influence patient compliance and overall nutritional status [66]. Temperature regulation is paramount to preventing exacerbation of mucosal sensitivity. Heat can exacerbate pain levels associated with conditions such as oral mucositis, which commonly manifests as oral ulcers, erythema, and hypersensitivity to temperature extremes [67]. Therefore, nutritionists should advise patients to avoid hot foods and beverages that could increase discomfort, promoting instead the consumption of items that are comfortably warm or at room temperature. This adjustment helps create a more tolerable eating experience, encouraging adequate nutrient intake. The dietary management plan should also strictly exclude known irritants to minimize discomfort during consumption. Acidic foods such as citrus fruits, tomatoes, and vinegar can sharply aggravate mucosal sensitivity, intensifying pain and potentially leading to further mucosal damage [68,69]. Spicy ingredients, including chili and pepper, should also be avoided, as the primary component—capsaicin—activates the TRPV1 pain receptor, causing a burning sensation that can hinder the swallowing process [69]. Similarly, rough or dry textures in foods such as toast and crackers can be abrasive to sensitive oral tissues, potentially causing tears or exacerbating existing wounds (see the irritant avoidance strategies in Figure 3). By systematically eliminating these thermal, chemical, and physical irritants, clinical nutritionists can significantly reduce the pain burden associated with eating. This approach not only enhances patient comfort but also serves a critical nutritional function: patients are far more likely to maintain adequate caloric intake when food does not provoke distress. The emphasis must shift toward identifying alternative flavors and soothing textures that allow for a safe, nourishing, and comparatively enjoyable eating experience [70,71].

## 6. Specific Dietary Interventions for Concurrent Toxicities (NIS)

**Figure 4:**
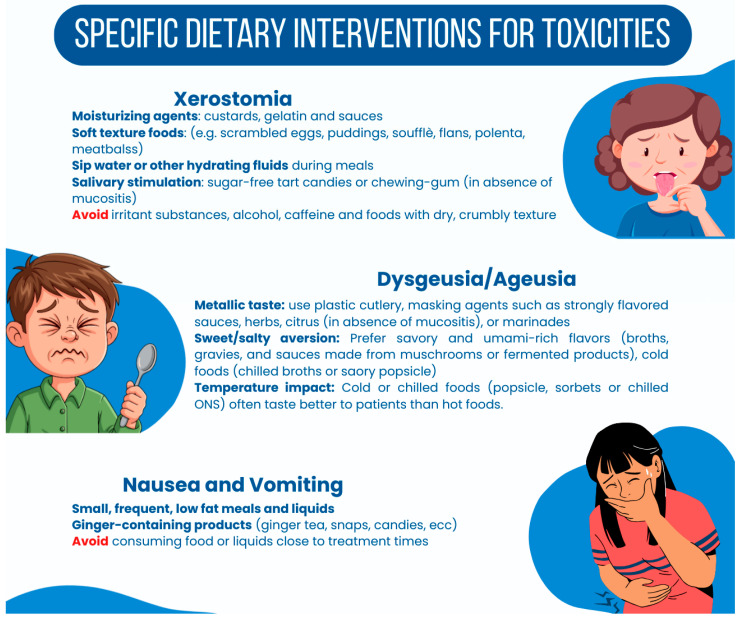
Specific Dietary Interventions for Concurrent Toxicities.

Targeted dietary interventions are essential for effectively managing the specific NIS that arise from disease burden and treatment toxicity. As already mentioned before, frequent complications occurring in patients undergoing treatment for HNC include xerostomia, dysgeusia, ageusia, nausea, and vomiting. In this context, incorporating specific dietary adjustments can greatly enhance patient comfort and nutritional intake (Figure 4).

### 6.1. Xerostomia

Xerostomia is defined as the subjective sensation of oral dryness, often accompanied by hyposalivation, an objective reduction in salivary flow. It represents one of the most prevalent and persistent complications in HNC, affecting the vast majority of patients undergoing radiotherapy, mainly due to the radiosensitivity of salivary glands. Utilizing high-moisture foods is essential for patients experiencing dry mouth. Foods such as custards, gelatin, and sauces can provide critical moisture while offering nutritional value. These additions not only lubricate the oral cavity but also create a cohesive bolus that reduces friction during the pharyngeal phase of swallowing [72,73]. To further mitigate the mechanical challenges, clinicians should recommend inherently soft, moisture-rich textures. Foods such as scrambled eggs, meatballs in gravy, puddings, and soufflés require minimal mastication and oral manipulation, promoting safer and less fatiguing swallowing. Furthermore, the “wash down” technique—sipping water or hydrating fluids concurrently with solid food—is critical; this practice compensates for the lack of natural saliva, facilitating bolus transit and preventing the sensation of choking [74] (Level of Evidence: Moderate/Expert Consensus). To stimulate residual salivary function, the use of sugar-free tart candies or chewing gum can be beneficial, provided there is no concurrent mucositis. These items are effective because they encourage the salivation, which can alleviate the sensation of dryness. Chewing gum, in particular, has been shown to enhance saliva flow rates and reduce self-reported symptoms of dry mouth [72,75]. However, it is essential to choose sugar-free options to minimize the risk of dental caries, especially since xerostomia increases the likelihood of oral infections and decay. Patients should be advised to avoid irritant substances that can exacerbate dryness and discomfort strictly. Alcohol and caffeine can contribute to dehydration and should be eliminated from their diets. Additionally, foods with dry, crumbly textures—such as dry bread, chips, and crackers—should be avoided as they can be particularly harsh on a sensitive oral mucosa, causing further irritation and challenges during swallowing [75]. By guiding patients to modify their diets accordingly, clinicians can help reduce discomfort and enhance overall nutritional quality.

### 6.2. Dysgeusia/Ageusia

Dysgeusia (taste distortion) and ageusia (loss of taste) are frequent and distressing complications of HNC treatment, resulting from radiation-induced damage to the taste buds (gustatory papillae) and neurotoxicity from chemotherapy agents. These alterations can manifest as “phantom” tastes—most commonly metallic or bitter—or a generalized blunting of flavor, which significantly contributes to food aversion and malnutrition. To mitigate this, recommending the use of plastic cutlery instead of metal utensils can help to reduce the ionization that exacerbates this sensation. Additionally, masking agents such as strongly flavored sauces, herbs, or marinades can enhance the overall flavor of foods. These flavor enhancers can distract the palate from unpleasant tastes, making the meal more appealing and palatable [74,76]. Innovative approaches, such as incorporating citrus or vinegar-based marinades, are also effective, provided that they do not cause irritation or discomfort due to concurrent mucositis. In addition, patients often report aversions to sweet and salty flavors during treatment. To address these aversions, utilizing savory and umami-rich flavors can be particularly beneficial. Broths, gravies, and umami-rich sauces made from mushrooms or fermented products can enhance the sensory profile of meals and encourage consumption. Cold options, such as chilled broths or savory popsicles, can also be explored as a refreshing and satisfying alternative [77]. The temperature at which foods are consumed can also significantly influence taste perception. Cold or chilled foods, such as sorbets, popsicles, or chilled ONS, often taste better to patients than hot foods (Level of Evidence: Low/Expert Opinion). The cooling sensation can distract from any unpleasant taste, making it a valuable strategy in dietary management during the acute phases of treatment. Encouraging patients to explore various textures and temperatures will enable them to identify appealing options that satisfy their nutritional needs while remaining aware of their altered taste perceptions [78]. Recent literature emphasizes the importance of early gustatory profiling and targeted flavor masking to improve oral tolerance [79].

### 6.3. Nausea and Vomiting

Managing nausea and vomiting in patients undergoing HNC treatments requires tailored dietary adjustments to enhance gastric tolerance and improve overall well-being. The cornerstone of this strategy is the “Small and Frequent” principle: consuming 6–8 small, nutrient-dense meals daily rather than the traditional three large meals [80].

Large volumes of food cause gastric distension, a potent vagal stimulus that can exacerbate nausea. This approach helps minimize gastric load and discomfort, as larger meals can aggravate nausea. Encouraging patients to eat smaller portions several times throughout the day improves tolerability. It can prevent overwhelming sensations associated with larger meals, helping prevent malnutrition due to decreased oral intake during treatment. Studies have indicated that a variety of dietary interventions can improve tolerance in cancer patients experiencing nausea [81]. The use of ginger-containing products can serve as a complementary approach to symptom relief in the management of nausea, primarily through their antagonism of 5-HT3 receptors in the gut. Ginger has been widely recognized for its antiemetic properties, and incorporating ginger tea, ginger snaps, or ginger candies into the patient’s diet can be beneficial. These items not only provide flavor but also may have a soothing effect on the gastrointestinal tract, supporting the management of nausea (Level of Evidence: Moderate). Research indicates that ginger can reduce the severity and duration of nausea, particularly in the context of chemotherapy and related conditions [82]. It is also crucial to consider the timing of food intake. Patients should avoid consuming food or liquids near treatment times to reduce the risk of nausea. Additionally, ensuring compliance with prescribed antiemetic medications is essential, as these treatments are designed to prevent nausea and vomiting effectively. Recent studies have shown that even with advances in pharmacological agents, a significant percentage of patients still experience recurring nausea and vomiting, underscoring the importance of dietary interventions in complementing medical treatments [81].

The global nutritional strategies and symptom-driven dietary adjustments for managing HNC-related toxicities are summarized in the operational toolkit provided in Table 1.

## 7. Discussion

The clinical burden of dysphagia—often exceeding 50% even prior to treatment initiation and escalates precipitously during chemoradiotherapy—and malnutrition in HNC patients presents a uniquely demanding clinical landscape, characterized by the convergence of anatomical obstruction and treatment-induced toxicity [83,84]. This functional decline is not merely a quality-of-life issue, representing a critical survival determinant. Indeed, dysphagia and associated NIS are directly correlated with severe adverse outcomes, including aspiration pneumonia, treatment interruptions, and severe malnutrition, necessitating a shift from reactive symptom management to proactive nutritional surveillance [85]. Standardized malnutrition assessments, such as the MUST and NRS-2002, along with the application of the GLIM diagnostic criteria, are crucial for early identification of patients at risk of malnutrition. These instruments allow for targeted interventions that can significantly improve patient outcomes [86]. The incorporation of functional assessments of swallowing, such as VFSS and FEES, provides an evidence-based foundation for developing individualized dietary plans that accommodate specific swallowing capabilities, facilitating greater oral intake and reducing the risk of complications. The strategies outlined in this paper—ranging from High-Energy/High-Protein (HE/HP) fortification to specific symptom management protocols—represent a shift towards “adaptive nutrition.” However, it must be acknowledged that this proposed framework relies heavily on a synthesis of expert opinion and existing frameworks (ESPEN, IDDSI), rather than direct prospective clinical validation. By employing particular texture modifications for odynophagia or “flavor masking” techniques for dysgeusia, clinicians could maintain oral intake longer into the treatment course [87,88]. By fortifying foods and employing the IDDSI framework, clinicians could offer personalized dietary strategies tailored to each patient’s unique needs, optimizing caloric and protein intake while ensuring safety and palatability. Beyond the physiological challenges, the management of HNC must address the profound psychological burden of eating. Food is inherently social and emotional; its reduction to a source of anxiety or pain can be devastating. Patients often experience “social dysphagia”—the embarrassment or fear of choking, coughing, or taking too long to eat in the presence of others—leading to social isolation and depression. Furthermore, ageusia and the mechanical nature of tube feeding can strip eating of its hedonic value. Effective management requires acknowledging this grief. Clinicians should actively involve family caregivers as “coaches” rather than “enforcers” to reduce mealtime conflict. Early referral to psycho-oncology support or patient support groups is essential to help patients navigate the loss of their “eating identity” and maintain the motivation required for rehabilitation.

Overall, a multi-disciplinary approach, coordinated among clinical nutritionists, speech-language pathologists, and oncologists, is paramount for effectively managing the nutritional needs of HNC patients. By proactively addressing the specific challenges posed by the treatment and the disease itself and implementing evidence-based nutritional strategies, clinicians could significantly improve both the nutritional status and quality of life for these patients during and after their cancer treatment.

## 8. Conclusions

This paper aimed to propose a comprehensive, evidence-based conceptual framework tailored to the challenges posed by treatment and disease-related conditions in HNC patients. The integration of systematic nutritional assessments, functional swallowing evaluations, and targeted dietary interventions may significantly improve the quality of care for these patients. By emphasizing high-energy, high-protein dietary fortifications and employing standardized guidelines such as the IDDSI framework, clinicians can effectively address the multifactorial nature of malnutrition and dysphagia prevalent among HNC patients. Future research should therefore prospectively explore the application of this framework across diverse oncology centers, evaluating feasibility, patient adherence, and clinical treatment outcomes. Randomized controlled studies are needed to determine if the framework can be validated as a standardized component of multidisciplinary cancer care involving clinical nutritionists, dietitians and oncologists.

## Figures and Tables

**Table 1:** Conceptual Framework for Nutritional Management and Symptom-Driven Dietary Adjustments in Head and Neck Cancer Patients.

Category	Clinical Recommendations	Practical Strategies & Tips
**Nutritional** **Requirements**	***Targets per day:***	Prioritize high-energy, high-protein (HE/HP) oral fortification. Add skim milk powder, cream cheese, or neutral oils (e.g., olive, canola) to meals to increase density without adding volume.
	Protein: 1.2–2.0 g/kg	
	Energy: 25–30 kcal/kg	
	Water: 30–35 mL/kg	
**Texture** **Modification** **(IDDSI)**	***Food:***	
	**Levels 7–6** Maintain moisture **Levels 5–4** Eliminate lumps/husks	**Levels 7–6** Integrate sauces, gravies, and fats; sip fluids concurrently with solids **Levels 5–4** Finely chop and moisten (L5) or blend/sieve (L4) for smooth consistency
	***Liquids:***	
	**Levels 2–3** Thickened consistency	Use thickening agents and visual tests like the spoon-tilt test for safety
**Pain** **Management**	**Thermal Control** Avoid extreme temperatures	Serve food and beverages at lukewarm or room temperature to avoid aggravating mucositis.
	**Irritant Avoidance** Minimize chemical/physical triggers	Avoid: Acidic foods (citrus, tomato, vinegar), spicy ingredients (chili), and rough textures (toast, crackers).
**Oral** **Nutritional** **Supplements** **(ONS)**	**Dosing** 1–2 servings per day	Targets: Aim for 300–600 kcal and 15–30 g protein per serving.
	**Composition** Standard or disease-specific	Timing: Administer between meals or as a preload to avoid the “displacement effect” on regular food intake.
**Management of** **Xerostomia**	**Moisture Retention** Use high-moisture foods	Focus on moisturizing agents: custards, gelatin, sauces, and gravies Soft textures: scrambled eggs, meatballs in gravy, puddings Use sugar-free tart candies or chewing gum (if no mucositis)
	**Salivary Stimulation** Encourage flow.	
**Management of** **Dysgeusia/Ageusia**	**Flavor Masking** Counteract metallic/bitter tastes	Use plastic cutlery to reduce metallic aftertaste. Masking: Strongly flavored sauces, herbs, and marinades. Flavor Aversions: Prefer savory/umami (broths, mushrooms) and cold/chilled foods (sorbets, popsicles).
	**Sensory Enhancement** Use umami-rich profiles	
**Management of** **Nausea & Vomiting**	**“Small & Frequent”** 6–8 small meals daily	Avoid eating close to treatment times. Use ginger-containing products (tea, candies, snaps) for natural antiemetic support. Focus on low-fat meals to improve tolerability.
	**Gastric Load Reduction** Minimize vagal stimulus	

## Data Availability

Not applicable.

## Abbreviations

The following abbreviations are used in this manuscript:

HNC	Head and Neck Cancer
NIS	Nutritional Impact Symptoms
ONS	Oral Nutritional Supplements
IDDSI	International Dysphagia Diet Standardization Initiative
MUST	Malnutrition Universal Screening Tool
NRS-2002	Nutritional Risk Screening 2002
GLIM	Global Leadership Initiative on Malnutrition
HE	High Energy
HP	High Protein
